# Implementation of Outpatient Automated Stewardship Information System (OASIS©) Audit and Feedback in Two Healthcare Systems

**DOI:** 10.1017/ash.2024.175

**Published:** 2024-09-16

**Authors:** Christine MacBrayne, Amy Keith, Nicole Poole, Cory Hussain, Joshua Tucker, Timothy Jenkins, Theresa Morin, Holly Frost

**Affiliations:** Children’s Hospital Colorado; Denver Health and Hospital Authority; Denver Health; Denver Health Medical Center

## Abstract

**Background:** Combating antibiotic resistance, exacerbated by widespread unnecessary outpatient antibiotic prescriptions, necessitates innovative stewardship solutions. Audit and feedback reports are effective but often resource heavy. We introduced a free, open-source system, Outpatient Automated Stewardship Information System (OASIS©), for automating the creation and distribution of recurring audit and feedback reports to clinicians to improve antibiotic prescribing. **Methods:** We used mixed methods to evaluate implementation of OASIS© across 11 clinics at Denver Health and Hospital Authority (DHHA) and Children’s Hospital Colorado (CHCO) from July 2022 to August 2023. Both sites host their own Epic® electronic healthcare and enterprise data warehouse systems. R statistical software was utilized to retrieve and process the data needed to create individual[HCM1] clinician audit and feedback reports with peer comparison. Reports were provided for 1) antibiotics prescribed for respiratory diagnoses, 2) antibiotics prescribed for respiratory diagnoses where antibiotics are never indicated, 3) first-line antibiotic prescribing for acute otitis media (AOM), and 4) five-day duration of antibiotics for children two years and older with AOM. Feedback reports for each metric were emailed to clinicians for three consecutive months. The primary outcome was adaptations needed to implement OASIS©. Secondary outcomes included fidelity (measured by email readership), time to set up and maintain the program, and barriers and facilitators to implementation (assessed by four qualitative interviews with OASIS© stakeholders). **Results:** The most significant adaptations made pertained to the automation of OASIS© reports for organizations not using R for data retrieval and reporting, setting up OASIS© specific email addresses, and validating clinician fidelity via read receipts. Fidelity was higher at DHHA (91-100%) compared to CHCO (10-30%). When interviewed, data analysts expressed that time for initial setup ranged from 1-6 hours. After reporting was automated, the estimated monthly time to send reports was 10 minutes. Views on setup complexity were split, but all recognized the readability of the reports and OASIS©’s value for improving prescribing behaviors. The greatest barriers to implementation included obtaining analytic resources for initial setup and the need to download additional R packages. No interviewee had prior experience creating audit and feedback reports. **Conclusions:** Implementing OASIS© requires addressing system diversity and knowledge gaps in outpatient informatics and antibiotic stewardship. Despite these challenges, the tool proved efficient and beneficial for monitoring and reporting antimicrobial prescribing. This free tool could likely be effectively disseminated to other health systems given the limited time and resources required for adaptations, setup, and monitoring. [HCM1]I

